# Comparing Sanger sequencing and high-throughput metabarcoding for inferring photobiont diversity in lichens

**DOI:** 10.1038/s41598-018-26947-8

**Published:** 2018-06-05

**Authors:** Fiona Paul, Jürgen Otte, Imke Schmitt, Francesco Dal Grande

**Affiliations:** 1 Senckenberg Biodiversity and Climate Research Centre (SBiK-F), Senckenberganlage 25, 60325 Frankfurt am Main, Germany; 2 Institute of Ecology, Evolution and Diversity, Goethe University Frankfurt am Main, Max-von-Laue-Str. 9, 60438 Frankfurt am Main, Germany; 3 Departamento de Farmacología, Farmacognosia y Botánica, Universidad Complutense de Madrid, 28040 Madrid, Spain

## Abstract

The implementation of HTS (high-throughput sequencing) approaches is rapidly changing our understanding of the lichen symbiosis, by uncovering high bacterial and fungal diversity, which is often host-specific. Recently, HTS methods revealed the presence of multiple photobionts inside a single thallus in several lichen species. This differs from Sanger technology, which typically yields a single, unambiguous algal sequence per individual. Here we compared HTS and Sanger methods for estimating the diversity of green algal symbionts within lichen thalli using 240 lichen individuals belonging to two species of lichen-forming fungi. According to HTS data, Sanger technology consistently yielded the most abundant photobiont sequence in the sample. However, if the second most abundant photobiont exceeded 30% of the total HTS reads in a sample, Sanger sequencing generally failed. Our results suggest that most lichen individuals in the two analyzed species, *Lasallia hispanica* and *L. pustulata*, indeed contain a single, predominant green algal photobiont. We conclude that Sanger sequencing is a valid approach to detect the dominant photobionts in lichen individuals and populations. We discuss which research areas in lichen ecology and evolution will continue to benefit from Sanger sequencing, and which areas will profit from HTS approaches to assessing symbiont diversity.

## Introduction

High-throughput sequencing (HTS) technologies have revolutionized the way we study microbial diversity and other complex ecological communities^1,2^. In particular, DNA metabarcoding - the identification of organisms from environmental samples using HTS of standardized DNA barcodes - has allowed rapid and cost-effective taxonomic assessments of a wide range of microbial groups from a variety of habitats in marine and terrestrial ecosystems^3^. Metabarcoding has also advanced our understanding of the distribution, abundance, and community structure of both symbiotic and free-living microorganisms^4–6^. Because HTS of DNA barcodes, e.g. on the Illumina MiSeq, Ion Torrent, or PacBio platforms, allows taxonomic assessment at a far greater depth and resolution than conventional Sanger sequencing^2,7^, it also facilitates discovery of rare taxa and detection of previously unrecognized eukaryotic and prokaryotic microbiomes^8–12^. Studying this vastly unexplored microbial biosphere is one of the new frontiers of microbial ecology, currently changing our understanding of species interactions and their ecological and evolutionary dynamics^13^.

One of the most striking examples of the HTS-driven paradigm shifts comes from lichen symbiosis research^14–16^. For more than a century, lichens have been viewed as a symbiotic association between two, maximally three partners, i.e. a fungus (the mycobiont) and a green alga and/or cyanobacterium (the photobiont). DNA sequences supported this notion: in most cases researchers detected a single species of mycobiont and photobiont in a thallus based on unambiguous Sanger sequence electropherograms. In recent years, HTS-based studies have uncovered a tremendous species diversity associated with lichen symbioses (e.g.^17–19^). Lichen thalli harbor hyper-diverse bacterial as well as fungal communities (e.g.^16,20^). This previously unrecognized lichen-associated biosphere may act as a significant functional component of the symbiosis, supporting the growth and regeneration of the thallus, and modulating the holobiont’s response to environmental triggers^21–23^.

Recent HTS studies on lichen photobiont diversity suggest that the presence of multiple, genetically differentiated algae within a single thallus is a common phenomenon in lichens^24–26^. This raises the question to what extent Sanger-based studies underestimate within-thallus green algal diversity, and whether the single Sanger sequence obtained from an individual corresponds to the most abundant photobiont in that individual^27^. These assumptions, however, have never been formally tested.

In this study, we compare HTS and Sanger sequencing methods for estimating the diversity of lichen-associated green algal photobionts. In particular, we address the following questions: (i) Are the photobionts identified via Sanger sequencing always the most abundant taxa in the thallus as inferred by HTS? and (ii) If more than one photobiont is present in a lichen individual: Is there an abundance threshold that precludes the generation of an unambiguous Sanger sequence?

## Results

### Overall sequencing success

From the Sanger sequencing of the 240 samples, we obtained 183 (full length ITS) and 198 (ITS2) high quality sequences. This corresponds to a sequencing success rate of 76.25% (full length ITS) and 82.5% (ITS2) (Supplementary Table S1). The excluded, low quality sequences were partially sequences containing many ambiguous bases, which reflected variable positions distinguishing the different OTU representative sequences obtained from the Illumina metabarcoding study. We got blast hits of >99% identity to one single OTU representative sequence for all of the Sanger sequences of the full ITS region. In the set of Sanger sequences amplified with the metabarcoding primers covering only ITS2, 99% of sequences were also clearly assignable to a single Illumina metabarcoding OTU. However, one Sanger sequence matched two OTUs at 100% sequence similarity and one Sanger sequence had a highest hit of 98.27% sequence similarity. Both sequences could not be assigned to an OTU, since they contained ambiguous bases at several variable sites that are used to determine OTU membership. We therefore excluded them from all downstream analyses.

### Sanger sequencing retrieves most abundant Illumina OTU

Each Sanger sequence (full length ITS, and ITS2-only) that passed the quality filtering corresponded to the most abundant OTU in the HTS metabarcoding dataset (Supplementary Table S2). There was not a single exception to this pattern. This also held true for samples, which had OTU6 as the most abundant algal partner. OTU6 was excluded in the Illumina metabarcoding study^25^ because it was present in less than 5% (n = 12) of the total number of samples. In all of the five cases, in which OTU6 was found to be the predominant photobiont, the Sanger sequences of both datasets (full length ITS and ITS2) were assigned to OTU6.

### Sanger sequencing fails when the second most abundant alga reaches 30% abundance

**Figure 1:**
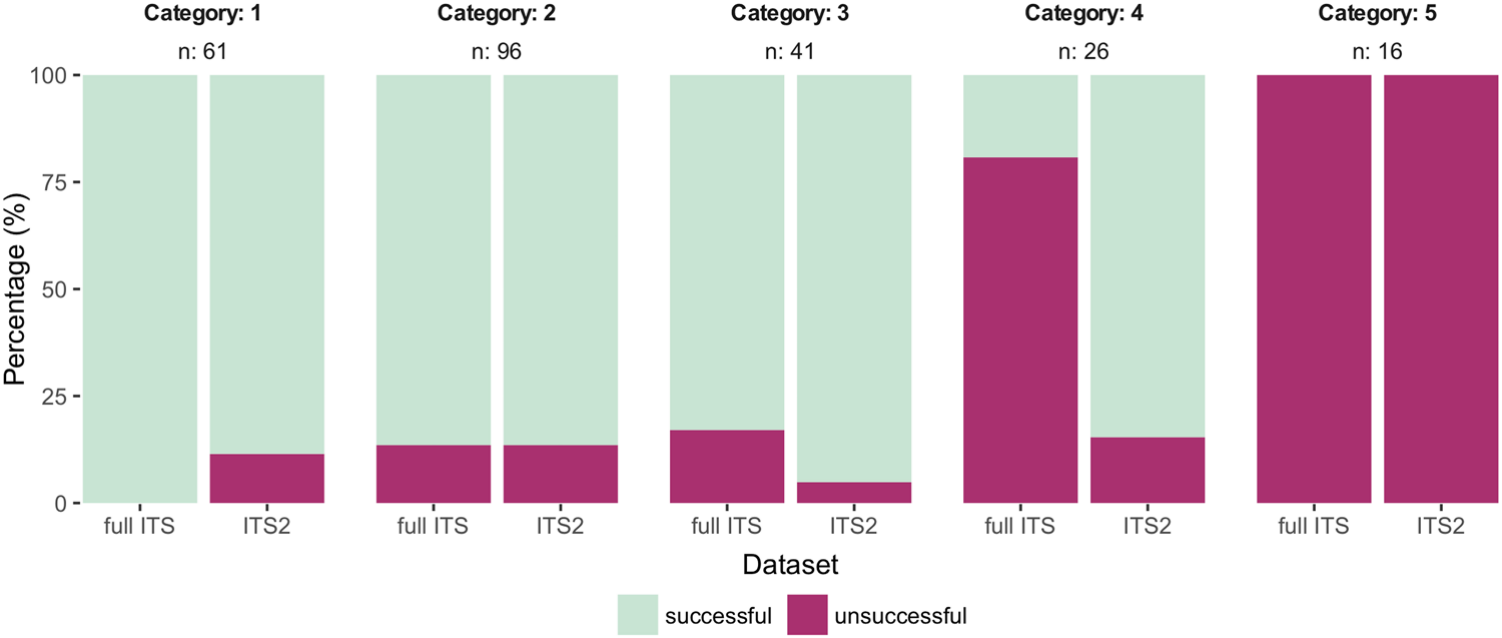
Sanger sequencing success depending on the abundance of the second most abundant photobiont for the full ITS (left) and ITS2 (right) data sets. Abundance categories based on Illumina ITS2 metabarcording^24^ are defined as follows: *cat. 1*: only a single photobiont present; *cat. 2*: secondary photobiont accounts for up to 10% of sequence reads; *cat. 3*: secondary photobiont accounts for 10–20% of reads; *cat. 4*: secondary photobiont accounts for 20–30% of reads; *cat. 5*: secondary photobiont represented by more than 30% of sequence reads.

For each of the datasets, we investigated the influence of the frequency distribution of the Illumina metabarcoding OTUs of each sample on the quality of the obtained Sanger sequence. When the predominant photobiont represented 100% of Illumina reads in a sample, 100% of the full length ITS (61 sequences) and 88.52% of the ITS2 (54 sequences) Sanger sequences were of high quality (Fig. 1). The proportion of obtained high quality Sanger sequences decreased with an abundance increase of the second most abundant photobiont. This trend held true for the full length ITS and ITS2-only datasets. Once more than 30% of Illumina reads were assigned to the second most abundant photobiont OTU, 0% (0 sequences) of Sanger sequences were of high quality, while 100% (16 sequences) of Sanger sequences failed to pass previous quality control measures (Fig. 1, Supplementary Table S1).

### Diversity found in ITS1 compared to ITS2

The OTUs assigned to each sample based on the ITS1 sequence retained from the full ITS Sanger sequences always matched the OTU assignment based on the ITS2 sample. We found no differences in diversity between ITS1 and ITS2 for the *Trebouxia* algae associated with the two lichen species, *Lasallia pustulata* and *L. hispanica*. For each sample, the OTU assigned based on the ITS1 region also coincided with the predominant photobiont found in the HTS data (Supplementary Table S3).

## Discussion

While the implementation of HTS technologies has greatly advanced our understanding of the hyper-diverse bacterial and fungal microbiomes integral to the lichen symbiosis^20^, most of the taxonomy and diversity estimates of lichen photobionts are still based on conventional Sanger sequencing of a single DNA barcode, the internal transcribed spacer (ITS) rRNA. This is evident from the vast number of ITS sequences of *Trebouxia* spp., the most common green algal genus of lichenized algae, deposited in NCBI GenBank (>6,000 algal sequences from more than 100 lichen genera; accessed in January 2018). Sanger sequences of lichenized algae obtained via amplification and sequencing of mixed symbiont DNA from lichen thalli have been used not only for molecular identification and phylogenetic analysis of photobionts^28–30^, but also for inferring fungal-algal association patterns^31–33^, and for reconstructing photobiont phylogeography and ecological niches^34,35^. The presence of multiple photobionts within a single thallus^24,25,27,36–40^, however, raises the question whether Sanger-based datasets are reproducible and biologically meaningful. In this study, we compared Sanger-based identification and diversity estimates of photobionts with photobiont abundances observed in the same DNA extracts inferred via Illumina MiSeq HTS of the PCR products using the same primers.

### Sanger-based and HTS datasets inform each other

Our results clearly indicate that the photobiont identified by Sanger sequencing always corresponds to the most abundant photobiont in a sample. Furthermore, our results help to verify the accuracy of the HTS sequence filtering steps utilized in^25^. One of the six photobionts (OTU6) identified via Sanger sequencing was excluded from the HTS photobiont dataset in^25^, because it was present in less than 5% of the samples (although it was the predominant alga in some samples). The consistent recovery of this OTU using Sanger sequencing suggests that, in order to avoid excessive filtering and keep biologically relevant units in the analysis, the filtering of the HTS OTU table should retain OTUs if they represent the predominant alga in at least one sample.

One limitation that has been attributed to HTS platforms is the limited sequence length (<500 bp)^7^. In our study the addition of the ITS1 portion did not improve taxonomic resolution: the OTUs defined via the full ITS barcode were always concordant with the OTUs based on ITS2. Thus, in the case of the *Trebouxia* photobionts analyzed in this study, all belonging to the *Trebouxia* clade S (*simplex*/*letharii*/*jamesii* sensu^41^), the implementation of long read sequencing platforms, such as PacBio or Ion Torrent, are not expected to improve species discrimination and phylogenetic information.

### Most lichen individuals contain a single algal population

The performance of Sanger sequencing degraded considerably when the second most abundant photobiont in a sample increased in relative abundance, and Sanger sequencing failed entirely when the second most abundant photobiont exceeded 30% of the total sample reads. This is particularly relevant if we consider that almost all algal ITS sequences deposited in public databases were obtained from direct PCR and sequencing of DNA mixtures from lichen thalli without any cloning steps. The inherent limitations of Sanger sequencing in obtaining readable electropherograms from samples with two abundant photobionts thus suggest that the vast majority of lichen thalli indeed host a single predominant algal population. If ITS Sanger sequencing fails because multiple algal strains are present in high abundances (>30% of total reads in sample), it may still be possible to obtain readable Sanger sequences from the sample by using lineage specific algal primers^27^. Our analysis suggests that each lichen individual contains maximally two, or very rarely three, abundant *Trebouxia* strains. Thus, Sanger ITS sequencing can be a viable approach to comprehensively sample green algal diversity, even if there is more than one abundant photobiont in a thallus.

### Which research questions can be addressed using Sanger sequencing?

The ability of Sanger sequencing to always, and unfailingly, detect the predominant algal species in a lichen thallus supports the continued implementation of this sequencing technique for addressing a wide variety of questions in lichen ecology and evolution. Specifically, Sanger ITS barcoding will continue to be useful in the following types of studies: (i) Assessing coevolutionary patterns in the lichen symbiosis: abundances and distributions of species exhibit often great stability on ecological timescales. Sanger sequencing of the ITS barcode marker allows for the rapid identification of the most common, i.e. biologically relevant, photobionts associated with a lichen. Sanger-derived photobiont databases can thus be used to study patterns of fungal-algal associations (e.g., generalism vs. specificity) across spatial and temporal scales, and for testing hypotheses of coevolution between fungi and their most common photobionts; (ii) Understanding symbiont-mediated niche expansion and conservation: according to the (bio)mass-ratio hypothesis, common species are disproportionately significant in ecosystem function^42–44^. Transposing the concept to lichen associations, the most abundant photobionts will numerically dominate lichen communities and thus play a disproportionately large role in the lichen community and ecosystem processes. Sanger-derived ITS photobiont datasets can thus be used to reconstruct ecological niches of the most common photobionts in a lichen community or population, and to study distributional trends of photobionts in response to environmental and/or anthropogenic cues. This is particularly relevant when assessing and predicting frequency and stability of fungal-algal interactions against a background of ongoing global change. Any change in the distribution of the most common species will be immediately significant for the entire community, because the most common species play important roles in engineering environments and supporting a large number of biotic interactions compared to rare species^45,46^.

### Which research questions can be addressed using high-throughput metabarcoding?

Because of its ultra-high-throughput and relatively low cost per read, Illumina MiSeq has become the method of choice for highly-multiplexed barcoding sequencing, i.e. the massive sequencing of hundreds, if not thousands, of samples in a single run. Additionally, and most importantly, HTS platforms allow the profiling of symbionts at the level of the whole community.

Thus, the most relevant application of the Illumina MiSeq (as any other HTS) platform is in the field of community ecology. Especially for species-rich communities, such as the lichen microbiomes, abundances of different taxa can vary considerably among different zones of a lichen thallus^26^, among individual hosts, as well as among different host taxa or habitats. As recently shown by^26^, only a combination of ultrastructural and HTS sequencing approaches on different parts of a lichen thallus (especially for species with foliose or fruticose growth form) and on many individuals will allow to fully uncover this potential variation^5^.

Rare species are increasingly recognized as potentially hidden drivers of microbiome function in various ecosystems^8,47,48^. This is especially true when rare or less abundant species strongly interact with more common species^46^. The role of relatively rare photobionts still needs to be investigated in lichens. HTS metabarcoding is thus the preferred method to obtain taxonomically more comprehensive symbiont datasets, as well as to investigate and model patterns of interactions among the photobiont taxa themselves within and among lichen thalli in various habitats. For instance, aspects of the insurance hypothesis^49^ could be tested using metabarcoding approaches. This hypothesis states that the presence of rare symbionts may buffer against environmental changes in order to maintain ecosystem function (in this case the survival of the lichen thallus) by allowing species turnovers, i.e. flexible abundance fluctuations in response to environmental changes. HTS platforms open up new avenues for investigating lichen symbioses and their complex microbiomes and will shed light on processes that may only be visible when the entire symbiont community is comprehensively considered.

## Methods

### Sampling

We collected populations of the lichen-forming fungi *Lasallia hispanica* and *L. pustulata* along an altitudinal gradient in the Sierra de Gredos mountain range in Central Spain. We sampled each species at six sites, and 20 individuals per site, resulting in a total collection of 240 individuals. Details on the study organisms, sampling, and sampling sites are given in^25^.

### Datasets

In the present study, we compared three datasets generated for each of the 240 individuals: (1) A metabarcoding dataset based on algal ITS2; (2) A Sanger sequence set based on the full length algal ITS; and (3) A Sanger sequence set based on ITS2 using the same primers as in the metabarcoding study.

### Metabarcoding dataset

Thallus pieces of ~8 mm in diameter were collected in proximity to the centre of each thallus^50^. We extracted total DNA using the CTAB method^51^, and amplified *Trebouxia* algal communities with algal-specific primers targeting the ITS2 region (FDGITS2-f: AGCGAAATGCGATACGTAGTGT; FDGITS2-r: GGGTGTTCTTGCCTGACCTC^25^). After library preparation the reads were paired-end sequenced (2 × 300 bp) on an Illumina MiSeq sequencer at StarSeq (Mainz, Germany). For a detailed account of Illumina sequencing results and bioinformatics processing of the reads please refer to^25^. Briefly, we quality-filtered, paired-end assembled, and demultiplexed the reads, before grouping them into operational taxonomic units (OTUs) using the de novo, single-linkage clustering algorithm SWARM v.2.0^52^. This OTU calling approach clusters sequences based on abundance information, as well as global sequence similarity. We excluded chimeric sequences and OTUs not assigned to the algal genus *Trebouxia* and filtered the OTU table for erroneous sequence occurrences as follows: we removed OTUs falling below 0.005% of the total read count in each sample, as well as OTUs present in less than 5% of samples (n = 12)^25,53–55^.

### Sanger sequencing (full length ITS rDNA)

We used the 240 DNA extracts described above to amplify full length algal ITS using the universal primers T1 and T4^56^. The PCR-reactions contained 0.65 U TaKaRa ExTaq (Clontech Laboratories Inc., Palo Alto, CA, USA), 2.5 µL buffer, 18.5 µL water, 0.5 µL bovine serum albumin (BSA, 10 mg ml^−1^), 2 µL dNTP mixture (2.5 mM each), about 5 ng total DNA (0.5 µL) and 0.22 µM (0.5 µL) forward and reverse primers. The following PCR conditions were used: initial denaturation at 95 °C for 4 min, followed by 35 cycles of 95 °C for 30 s, 63 °C for 20 s, 72 °C for 20 s and a final elongation for 5 min at 72 °C. We sequenced the amplicons using BigDye v3.1 chemistry (Applied Biosystems, Foster City, CA, USA) under the following cycle sequencing conditions: initial denaturation for 1 min at 95 °C, followed by 30 cycles of 96 °C for 10 s, 50 °C for 10 s, 60 °C for 2 min. The products were purified and sequenced on an ABI 3730 DNA Analyzer (Applied Biosystems).

### Sanger sequencing (ITS2)

From the 240 DNA extracts we also amplified the ITS2 region, using the same primers we used in the metabarcoding study (FDGITS2-f and FDGITS2-r). The PCR mixture as well as the cycling conditions were the same as described above for the full length ITS sequences. Sanger sequencing conditions were also the same as for the full length ITS fragments.

### Editing of Sanger sequences

Sanger sequences were manually evaluated in Geneious v9.1.7 (Biomatters Ltd., Auckland, New Zealand). In a first step, the forward and reverse sequences of each sample were paired-end assembled using the command De Novo Assembler with the Geneious Assembler, Highest Sensitivity and without trimming the sequences. If the assembly was unsuccessful without trimming the sequences, sequences were paired-end assembled allowing the sequences to be trimmed. Secondary peaks were automatically identified as peaks with a secondary peak height above 25% of the maximal peak. The assembled sequences were then manually evaluated and edited based on their electropherogram. We directly excluded sequences, for which the paired-end assembly failed and/or led to significantly shortened sequences. Further, we excluded sequences with ambiguous base calls and/or underlying peaks in the electropherogram that could not be resolved. Since it is impossible to perform robust taxonomic assignment for these sequences, we excluded them from the dataset and from further analyses, and we noted them as unsuccessful Sanger sequences. All sequences passing the quality filter were aligned in a multiple pairwise alignment with MAFFT version 7.308^57^ using default settings to further edit the sequences where needed. We then extracted the algal ITS1 (for the full length ITS dataset) and ITS2 (for both full length ITS dataset and ITS2 dataset) variable regions from the sequences using ITSx version 1.0.11^58^ with default settings.

### Comparison of datasets

To compare the Sanger sequence of each sample to the OTUs in the metabarcoding dataset, we ran a local nucleotide BLAST comparing each Sanger sequence to the representative sequences of the OTUs. We performed a local BLAST run using blastn v 2.2.30+^59^ with default settings, accepting sequence similarities of 95% and higher. We excluded sequences from further analysis, if there was (1) no clear BLAST hit to a single OTU representative sequence, i.e. the second-best BLAST hit was not significantly lower in score and identity than best BLAST hit and (2) the sequence similarity was not 99% or higher. If a Sanger sequence met these criteria, the matching OTU was assigned to the respective sample. We then checked whether the OTU assigned based on the Sanger sequence of each sample matched the most abundant OTU found in the metabarcoding dataset.

We examined whether the quality of the Sanger sequence was influenced by the abundance distribution of the OTUs in the metabarcoding dataset. We first split our dataset into the Sanger sequences of the full ITS sequence (amplified with universal primers T1 and T4) and the Sanger sequences of only ITS2 (amplified with primers FDGITS2-f and FDGITS2-r). Therefore, we assigned each sample to a frequency category based on the frequency of the second most abundant algal OTU in the metabarcoding dataset. We defined frequency categories as follows:

- category 1: most abundant alga accounts for 100% of sequences;
- category 2: secondary photobiont accounts for up to 10% of sequence reads;
- category 3: secondary photobiont accounts for 10–20% of reads;
- category 4: secondary photobiont accounts for 20–30% of reads;
- category 5: secondary photobiont represented by more than 30% of sequence reads.

We then checked which frequency categories resulted in a high-quality Sanger sequence. We calculated the proportions of samples with and without Sanger sequences in each of the categories and made stacked bar plots using R v. 3.3.3^60^ and the ggplot2 package^61^.

We compared the taxonomic resolution of ITS1 compared to ITS2, since we also had Sanger sequences covering the full ITS, from which we extracted the ITS1 region during the ITS extraction (see above). For this, we first clustered all ITS1 Sanger sequences at 99% sequence identity with vsearch version 2.4.3^62^ using the cluster_size command with default settings. The generated OTU representative sequences were then compared to the OTUs from the metabarcoding dataset. We compared the assigned OTUs based on ITS1 of each sample to the most abundant OTU from the metabarcoding dataset, noting whether they matched or not.

Sequence data are available in NCBI GenBank under accession numbers MH298924 - MH299121 and MH299122 - MH299304 for the ITS2 and the full ITS sequences, respectively.

## Electronic supplementary material

Supplementary Table S1

Supplementary Table S2

Supplementary Table S3
