# Author Correction: Comparing Sanger sequencing and high-throughput metabarcoding for inferring photobiont diversity in lichens

**DOI:** 10.1038/s41598-018-35358-8

**Published:** 2018-11-16

**Authors:** Fiona Paul, Jürgen Otte, Imke Schmitt, Francesco Dal Grande

**Affiliations:** 1 Senckenberg Biodiversity and Climate Research Centre (SBiK-F), Senckenberganlage 25, 60325 Frankfurt am Main, Germany; 2 Institute of Ecology, Evolution and Diversity, Goethe University Frankfurt am Main, Max-von-Laue-Str. 9, 60438 Frankfurt am Main, Germany; 3 Departamento de Farmacología, Farmacognosia y Botánica, Universidad Complutense de Madrid, 28040 Madrid, Spain

Correction to: *Scientific Reports* 10.1038/s41598-018-26947-8, published online 05 June 2018

In the “Supplementary Table S2” file originally published with this Article, columns E and G contained incorrect accession numbers. These errors have been corrected in the “Supplementary Table S2” file that now accompanies the Article.

